# Receptor for advanced glycation endproducts controls deleterious lung inflammation in severe *Pseudomonas aeruginosa *pneumonia in immunosuppressed mice

**DOI:** 10.1186/cc11701

**Published:** 2012-11-14

**Authors:** C Ravinet-Trillou, F Soubrier, A Caron, S Llopart, M Fontanie, F Lantreibecq, PE Rouby, C Menet, L Leotard, C Serradeil-Le Gal

**Affiliations:** 1 Sanofi, TSU ID, Toulouse, France; 2 Sanofi, SCP Biologics, Toulouse, France

## Background

The receptor for advanced glycation endproducts (RAGE/AGER) plays a key role in infections by sustaining tissue-damaging inflammation. This multiligand/multisignal pattern recognition receptor, overexpressed in lung and on innate immune cells, was shown to contribute to the pathogenesis of pneumococcal pneumonia and thus may be an attractive therapeutic target to treat severe pneumonia, one of the most common causes of death in western countries. Here, we characterized a new anti-human RAGE antibody (SAR) and its *in vivo *effect in a model of severe Gram-negative bacterial pneumonia in immunosuppressed mice.

## Methods

The SAR antibody was previously selected for binding affinity for the human sRAGE (Biacore) and for RAGE ligand binding inhibition (S100B, HMGB1, S100A6) by ELISA. A similar IC_50 _was found for SAR and anti-RAGE XT-M4 antibody (Pfizer/Wyeth) with HMGB1. Since SAR did not cross-react with mouse RAGE, transgenic C57Bl6 mice expressing human RAGE were engineered for *in vivo *testing. Mice, receiving at day 4 and day 1 cyclophosphamide for immunosuppression, were challenged with intranasal 10^7 ^or 10^8 ^colony forming units (CFU) *Pseudomonas aeruginosa *(PA01 strain) and treated 4 hours after with ciprofloxacin alone or in the presence of the selected RAGE antibody: survival, CFU and bronchoalveolar lavage (BAL) cytokines were measured. The anti-RAGE XT-M4 antibody was used as a reference.

## Results

In human RAGE KO/KI humanized mice, hRAGE expression was validated at the mRNA (RT PCR) and protein (IHC) levels. In the pneumonia model, higher loads of *P. aeruginosa *was used in transgenic versus wild-type mice to achieve more than 80% of mortality 24 hours after infection (10^8 ^CFU). XT-M4 antibody was injected at 15 mg/kg intravenously 4 hours after infection (alone or with ciprofloxacin, 0.1 and 0.3 mg/kg, intraperitoneal given 4 hours and 24 hours after infection). The association of XT-M4 and ciprofloxacin significantly increased survival compared with the antibiotic alone (ciprofloxacin 0.1/XT-M4 +54%, *P *< 0.066, ciprofloxacin 0.3/XT-M4 +78%, *P *< 0.01). The SAR antibody (7.5 and 15 mg/kg) also induced significant improvement in delaying mortality in transgenic mice when given in combination with ciprofloxacin versus antibiotic alone. Moreover, 30 mg/kg SAR with ciprofloxacin (10 mg/kg) reduced (but not significantly) BAL cytokine release (TNFα, KC, IL-12) 18 hours after infection.

## Conclusion

A survival benefit and partial control of inflammation was observed with anti-RAGE antibodies in combination with antimicrobial agent in a model of Gram-negative (*P. aeruginosa*) pneumonia in immunosuppressed mice. Thus, together with existing literature reports, these data are in favor of the contribution of RAGE to the pathogenesis of severe bacterial pneumonia.

